# Diabetic control and atypical antipsychotics: a case report

**DOI:** 10.1186/1752-1947-2-155

**Published:** 2008-05-14

**Authors:** Romina Lopez Gaston, Mohan George, Nangai Azhahan

**Affiliations:** 1 Psychiatric Intensive Care Unit, Queen Elizabeth Psychiatric Hospital (University Hospital), Birmingham, UK

## Abstract

**Introduction:**

People with schizophrenia are at increased risk of developing metabolic disturbances. This risk may be further exacerbated by the use of antipsychotic agents. Research is still ongoing to determine the metabolic impact of antipsychotics on glucose regulation. In this case report we review some of the possible mechanisms of action of antipsychotic medication on glucose regulation.

**Case presentation:**

We present the case of a 50-year-old man diagnosed with paranoid schizophrenia who developed type 2 diabetes mellitus whilst on treatment with second generation antipsychotics (SGA). His diabetes was controlled by a combination of antidiabetic drugs that were associated with his psychotropic treatment. Due to deterioration in his mental state, the patient was admitted on two occasions to a psychiatric unit during which his prescribed medication (olanzapine and risperidone) was discontinued and changed to aripiprazole. On both occasions, the patient suffered hypoglycaemic episodes and his antidiabetic treatment had to be adjusted accordingly. The patient did not require any antidiabetic treatment whilst on aripiprazole during the follow up period.

**Conclusion:**

Clinicians face regular dilemmas in trying to find the right balance between achieving control over a patient's mental illness and reducing any adverse effects associated with the prescribed medication. In patients receiving concomitant antidiabetic therapy, caution should be exercised when changing from one SGA to another. Whilst more longitudinal data are required, a trial of alternative SGAs, including aripiprazole in those developing type 2 diabetes and impaired glucose tolerance may be a worthwhile therapeutic option.

## Introduction

Second generation antipsychotics (SGAS) have been adopted as first line treatment for people with schizophrenia [1]. This has been based on a superior safety profile with regards to adverse events such as extrapyramidal symptoms in comparison to first generation (or 'conventional') antipsychotics [2]. However, many studies have provided convincing evidence for a high risk of metabolic abnormalities associated with the use of some of these agents [3]. These are of major concern owing to the additive effect on morbidity and mortality in a population with already increased prevalence of obesity, type 2 diabetes mellitus and cardiovascular disease [4].

The prevalence of diabetes mellitus among people with schizophrenia is approximately two to four times higher than in the general population, and schizophrenia appears to be an independent risk factor for diabetes mellitus [5]. The development of metabolic abnormalities within an individual patient can depend on the contribution of drug effects (for example, weight gain) as well as individual host factors, such as race, family history, disease or lifestyle. The part played by antipsychotic medication in the development of diabetes mellitus is an ongoing focus of research. Apart from increased adiposity, there are still unanswered questions about the mechanism of action involved in altering insulin sensitivity or secretion by these agents [5,6].

We present a patient with paranoid schizophrenia who developed type 2 diabetes whilst on treatment with SGAS. He suffered hypoglycaemic episodes on two separate occasions when the treatment was changed to aripiprazole with no change to his antidiabetic treatment.

## Case presentation

The patient, a 50-year-old Asian man diagnosed with paranoid schizophrenia when in his late twenties, had been treated with a variety of first generation antipsychotics for several years. Whilst an inpatient at age 42 years, he had been prescribed SGAS. He was medically fit, apart from suffering from hypertension controlled with perindropril 4 mg/day. He was discharged once clinically stable on olanzapine 20 mg/day. His records revealed that at age 48 years, whilst on 20 mg olanzapine, his random glucose levels were 17.6 and his glycosylated haemoglobin was 10.9%. He was started on oral hypoglycaemic agents by his general practitioner, a combination of gliclazide MR 30 mg/day and metformin 500 mg twice daily, which was later increased to 1 g twice daily because of poor response.

His diabetes mellitus was under control, but as his mental state continued to deteriorate, olanzapine was increased to 25 mg/day and subsequently changed to aripiprazole reaching a dose of 20 mg/day. After 2 months, the patient experienced gradual weight loss associated with episodes of hypoglycaemia. Gliclazide was consequently discontinued and he remained on metformin 1 g twice daily.

The patient was re-admitted to hospital 7 months later owing to poor compliance with medication and exacerbation of his psychiatric symptoms. Whilst in the community, his general practitioner restarted his diabetic medication (gliclazide 30 mg/day and metformin 500 mg twice daily) and combined it with a lipid-regulating drug (simvastatin 20 mg/day) and antihypertensive treatment (amlodipine 10 mg/day and perindropril 4 mg/day). On admission, his fasting glucose levels were between 3.7 and 6 mmol/l and he was overweight with a body mass index (BMI) of 27. Risperidone was added to the medication regime reaching a dose of 6 mg/day. His fasting glucose levels were between 4.2 mmol/l and 6 mmol/l. After 3 months, despite controlling his psychiatric symptoms, risperidone use resulted in intolerable side effects in the form of urinary incontinence, therefore it was discontinued and aripiprazole restarted reaching a dose of 15 mg daily. Six weeks later, the patient presented with symptomatic episodes of hypoglycaemia with fasting glucose levels between 2.1 and 3 mmol/l with no changes in body composition or other metabolic parameters. In consultation with his diabetologist, the oral hypoglycaemic medication was discontinued. The patient remained physically well with fasting glucose levels within normal range (3.5 to 6 mmol/l) during the 6 months that he remained in the unit.

## Discussion

The majority of studies indicate that SGA drugs which induce more weight gain (for example, clozapine and olanzapine) are associated with increased risk of diabetes mellitus and interpretations in the literature in relation to specific differences among these drugs have been controversial [7]. The increased prevalence of abnormalities in glucose regulation (for example, insulin resistance) and under-diagnosis of type 2 diabetes in patients with schizophrenia, prior to the commencement of antipsychotic medication, is a confounding factor [4,6]. In addition, the issue is complicated by the nature (mostly retrospective) and heterogeneity of the data with studies funded primarily by pharmaceutical companies [6,7].

Differing weight gain risk across the SGA agents seems to run alongside the variation in relative risk for metabolic disturbances [7]. One of the proposed mechanisms appears to be related to a greater H1 histamine receptor affinity associated with complex interplay among many other receptors (alpha1, H1, muscarinic, 5 hydroxytryptamine type 2A-2C and so on). Increased adiposity is linked to decrease in insulin sensitivity and changes in plasma glucose and lipid levels [6,8]. Emerging evidence in animal studies suggests that direct drug effects on beta-cell function and insulin action could be involved as factors independent from changes in body composition in up to a quarter of treatment-related new onset diabetes [7,9]. Rapid induction of hyperglycaemia sometimes accompanied by ketoacidosis has been reported in patients on clozapine and olanzapine without weight gain [9]. It seems likely that the hepatic insulin resistance develops with acute dosing, whereas weight gain and hyperlipidemia occur following repeated dosing. An alternative mechanism is inhibition of glucose transport into peripheral tissues, with suppression of cholinergic-stimulated insulin secretion by direct action on the pancreas, which involves antagonism of muscarinic M3 on beta-cells [6,9]. In addition, it is likely that the central nervous system plays an important role through the hypothalamus and its action over sympathetic and parasympathetic pathways on glucose regulation [6,8].

Data on the metabolic impact of aripiprazole suggest that it has little or no detrimental effect relative to other SGAs. However, two cases of diabetic ketoacidosis were reported in people with schizophrenia after starting aripiprazole [10-12] and further reports claim that it may even have a favourable impact on metabolic parameters [12]. The debate is still ongoing as a result of fewer long-term data owing to the limited time this medication has been on the market.

Our patient, who has diabetes treated with oral hypoglycaemic agents, experienced changes in body composition such as gradual weight loss and reversal of metabolic parameters towards normal levels associated with hypoglycaemia when the antipsychotic medication he was on was switched to aripiprazole for the first time. When his antipsychotic medication was changed to aripiprazole once again ten months later, following the discontinuation of risperidone owing to side effects, a second episode of hypoglycaemia occurred. On both occasions this led to review of his antidiabetic treatment. The hypoglycaemic episode could be explained by the discontinuation of olanzapine and/or risperidone as described in the literature [13] or the combination of oral hypoglycaemic medication with aripiprazole. However, it is relevant to note that the patient did not require any antidiabetic treatment whilst on aripiprazole during the follow up period.

## Conclusion

Clinicians face regular dilemmas in trying to find the right balance between achieving control over the patient's mental illness and reducing the adverse effects associated with the prescribed medication. Whilst research in the area is ongoing, psychiatrists should perform regular general health monitoring (including screening for diabetes mellitus) in all patients with schizophrenia. In patients receiving concomitant antidiabetic therapy, caution should be exercised when changing from one SGA to another. Whilst more longitudinal data is required, a trial of alternative SGAs, including aripiprazole in those developing type 2 diabetes and impaired glucose tolerance may be a worthwhile therapeutic option.

Further research is required to determine the potential of aripiprazole to prevent complications or reverse metabolic abnormalities in those patients with pronounced disturbances at baseline. If present, these properties could enhance treatment options in patients receiving concomitant antidiabetic therapy and in those patients with treatment resistance on combination therapy with drugs known to have a poor safety profile [14].

## Abbreviations

BMI: body mass index; SGA: second-generation antipsychotic.

## Competing interests

MG has received sponsorship from Janssen Cilag, manufacturers of risperidone, Otsuka Pharmaeuticals, manufacturers of aripiprazole, and Eli Lilly, manufacturers of olanzapine, for attending various conferences and has received honoraria from the same firms for speaking and chairing meetings. No other potential conflict of interest relevant to this article was reported.

## Authors' contributions

RLG, MG and NA participated in the sequence, alignment and drafted of the manuscript. All authors read and approved the final manuscript.

## Consent

Written informed consent was obtained from the patient for publication of this case report and any accompanying images. A copy of the written consent is available for review by the Editor-in-Chief of this journal.
